# Hybridization of surface plasmons and photonic crystal resonators for high-sensitivity and high-resolution sensing applications

**DOI:** 10.1038/s41598-022-25980-y

**Published:** 2022-12-09

**Authors:** Leila Hajshahvaladi, Hassan Kaatuzian, Maryam Moghaddasi, Mohammad Danaie

**Affiliations:** 1 Photonics Research Lab., Electrical Engineering Department, Amirkabir University of Technology, Tehran, Iran; 2 Faculty of Electrical and Computer Engineering, Semnan University, Semnan, Iran

**Keywords:** Optics and photonics, Physics

## Abstract

In this paper, an optical refractive index (RI) sensor based on a hybrid plasmonic-photonic crystal (P-PhC) is designed. In the sensor’s structure, some metallic rods are embedded in a rod-type photonic crystal (PhC) structure. Numerical simulations are performed based on the finite-difference time-domain (FDTD) method. The obtained results illustrate that the localized surface plasmons (LSP) induced by metallic rods can be excited in a PhC lattice to generate a hybrid P-PhC mode. According to the results, the hybrid mode provides unique opportunities. Using metallic rods in the coupling regions between waveguides and the resonant cavity significantly increases the interaction of the optical field and analyte inside the cavity. The simulation results reveal that high sensitivity of 1672 nm/RIU and an excellent figure of merit (FoM) of 2388 RIU^−1^ are obtained for the proposed hybrid P-PhC sensor. These values are highest compared to the purely plasmonic and or purely PhC sensors reported in the literature. The proposed sensor could simultaneously enhance sensitivity and FoM values. Therefore, the proposed hybrid P-PhC RI sensor is a more fascinating candidate for high-sensitivity and high-resolution sensing applications at optic communication wavelengths.

## Introduction

In recent years, optical refractive index (RI) sensors have been extensively studied due to the developing demands for sensing and detection applications^1–3^. The primary parameters to assess the sensing performance of RI-based sensors are sensitivity and figure of merit (FoM)^4–6^. The optical RI-based sensors are sensitive to small RI versions of the analyte. In plasmonic sensors, it originates from the interaction of the evanescent field with the analyte^7^. A perfect RI-based sensor should not only be highly sensitive to small RI versions but also needs to have a large FoM. In this regard, plasmonic^8–19^ and photonic crystal (PhC)^20–28^ structures have presented the most promising sensing abilities. Nevertheless, there are a few challenges for achieving the best sensing performance. Plasmonic-based RI sensors accentuate the light-matter interaction by exciting the surface plasmon polaritons (SPP) and localized surface plasmons (LSP) on the metal–dielectric interface^29–31^. These structures manipulate light within sub-wavelength scales^32,33^. However, the presence of metals in plasmonic-based RI sensors results in large ohmic losses and higher fabrication costs^34–37^. Therefore, plasmonic sensors usually tend to have better sensitivity. Albeit, they have a decreased FoM due to their higher losses. In contrast, PhC-based RI sensors tend to have lower losses and they are able to provide a higher FoM^38–42^. However, PhC sensors commonly exhibit a small field overlap with the analyte, ensuing in lower sensitivity as compared to plasmonic sensors. In PhC sensor structurs, the stability is investigated due to deviations which happen in the actual fabrication processes^43^.

Owing to the mentioned properties, one of the appealing and progressive techniques to acquire enhanced sensitivity and larger FoM is to combine a plasmonic component with a PhC structure to create an advanced hybrid plasmonic-photonic crystal (P-PhC) sensor^44–49^. In such a hybrid sensor structure, the nature of surface wave at the metal interface enhances the sensitivity and the lossless nature of the photonic crystal lattice enhances the FoM^50–52^. Therefore, combining both features can provide an outstanding sensing performance^5,53^. In recent years, the combination of a plasmonic component such as metal films or metallic nanostructures with a PhC structure has been explored and experimented^54–56^. The hybrid P-PhC sensor outperforms the individual plasmonic and PhC sensors. As well, the properties of the electromagnetic field in plasmonic and PhC structures are in lots of respects complementary in nature. It ends up enhancing the overall performance by such a hybridization. The hybrid sensor can expand the abilities of both plasmonic and PhC-based sensors via the simultaneous use of the strong light-matter interaction of the plasmonic component and the low losses of the PhC^57–59^. Furthermore, much less metal material is utilized in hybrid PhC-P sensors in comparison to purely plasmonic sensors which results in reducing the propagation losses and fabrication costs^5,60^.

In this paper, a hybrid P-PhC RI sensor is designed in which some metallic rods are embedded in a rod-type Si PhC structure. Using metallic rods in the coupling regions between the input and output waveguides and the resonant cavity significantly increases the interaction volume of the optical field and analyte inside the cavity. Increasing the light-analyte interaction in the cavity enhances the sensitivity and FoM of the sensor considerably. Here, the designed RI sensors are without labeling requirement. To further evaluate the overall sensing performance of the proposed sensor structure, we compare three RI sensor structures including the PhC sensor without metallic rods, the hybrid P-PhC sensor with two and four metallic rods in coupling regions. Then, the metallic rods parameters such as analyte region length, period and radius of metallic rods are investigated. Numerical simulations are performed based on the finite-difference time-domain (FDTD) method. A sensitivity of 1672 nm/RIU and a fairly high FoM of 2730 RIU^−1^ are achieved for the proposed hybrid P-PhC sensor. Based on the knowledge of the authors, these values are highest compared to the purely plasmonic and or purely PhC sensors reported in the literature. This paper is organized as follows: In “Sensing analysis of RI sensors” section, the theoretical basis of RI sensors has been presented. In “Theory and analysis of surface plasmon excitation in PhC structures” section, the theory analysis of SPP and LSP excitation in a PhC structure is investigated. In “The proposed hybrid P-PhC sensor's configuration” section, the proposed hybrid sensor’s configuration and analysis methods are described. In “Results and discussions” section, the simulation results are presented. Finally, “Conclusions” section is for conclusions.

## Sensing analysis of RI sensors

The optical refractive index (RI) of a medium is an important optical parameter in explaining light-matter interactions^61^. We will utilize two substantial parameters to characterize the sensing performance of optical RI sensors^50^:

- Sensitivity (S)
- Figure of merit (FoM)

**Figure 1:**
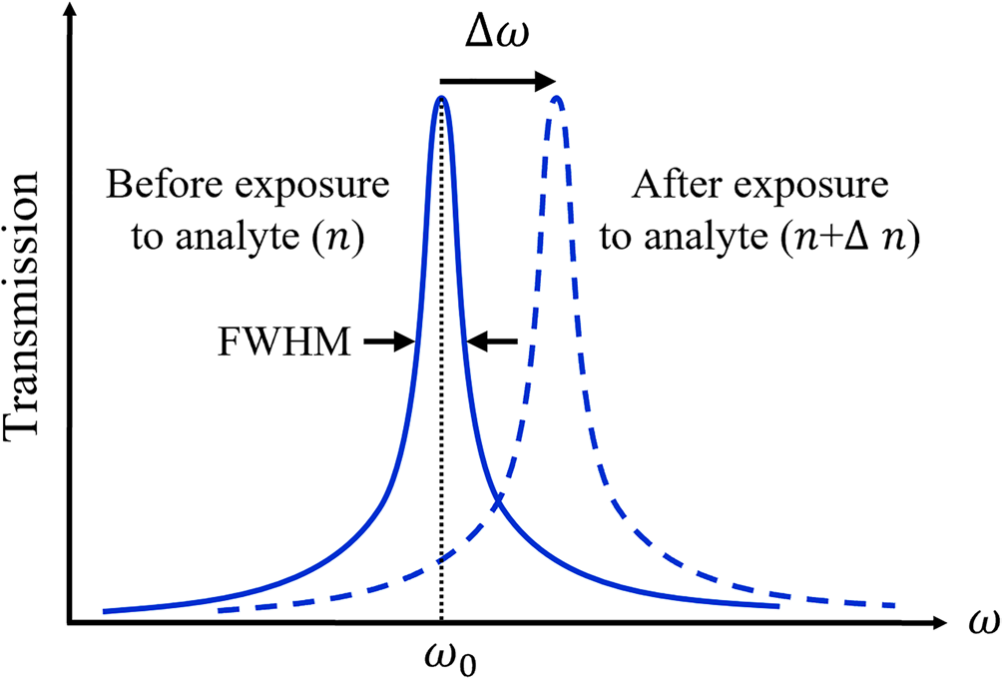
The sensing performance of an optical RI sensor.

All sensors have to be evaluated according to their sensitivity and FoM values. It is preferred that the values of both parameters be much higher. As illustrated in Fig. 1, in an optical RI sensor, varying RI of the analyte (${\Delta }n$) provides a shift in the resonance wavelength (${\Delta }\lambda$) and or frequency (${\Delta }\omega$) in the optical transmission response associated with the sensor.

Using the perturbation theory, the resonance frequency shift ($\Delta \omega$) can be determined as^20,62^:

1 $$\frac{{{\Delta }\omega }}{{\omega_{0} }} = - \frac{{\iiint {_{V}^{1} \left( {\delta \mu \left| {H_{0} } \right|^{2} + \delta \varepsilon \left| {E_{0} } \right|^{2} } \right)dV}}}{{\iiint {_{V}^{1} \left( {\mu \left| {H_{0} } \right|^{2} + \varepsilon \left| {E_{0} } \right|^{2} } \right)dV}}} = - \frac{1}{W}\iiint {_{V}^{1} \left( {\frac{\delta \varepsilon }{\varepsilon }.W_{e} + \frac{\delta \mu }{\mu }.W_{m} } \right)dV}$$

where $E_{0}$ and $H_{0}$ are the original electric and magnetic fields, respectively. $\mu$ and $\varepsilon$ are the permeability and permittivity, respectively. $W$ stands for the total energy stored in the resonant cavity, and $W_{e}$ and $W_{m}$ are the electric and magnetic energy densities, respectively. When $\mu$ does not change, Eq. (1) can be simplified as follows^20,53^:

2 $$\frac{\Delta \omega }{{\omega_{0} }} = - \frac{1}{W}\iiint {_{V}^{1} \left( {\frac{\delta \varepsilon }{\varepsilon } \cdot W_{e} } \right)dV = - \frac{1}{W}\iiint {_{V}^{1} \left( {\delta \varepsilon_{r} \varepsilon_{0} \left| {E_{0} } \right|^{2} } \right)dV}}$$

where $\varepsilon_{0}$ is the permittivity of the free space, $\varepsilon_{r}$ is the relative permittivity ($\varepsilon_{r} = n^{2} /\mu_{r}$) and $\mu_{r}$ is the relative permeability. From Eq. (2), the optical resonance frequency shift (${\Delta }\omega$) can be approximated by ${\Delta }\omega /\omega_{0} = - \sigma \left( {\delta n/n} \right)$, where $\sigma$ depends on the part of the resonance mode’s energy stored in the analyte region, and $\delta n$ is the RI variation. Thereby, the spectral sensitivity (${\text{S}}$) of the optical RI sensor, which provides the resonance wavelength shift (${\Delta }\lambda$) for a given $\delta n$ at the resonance wavelength ($\lambda_{0}$) can be expressed as follows^53^:

3 $${\text{S}}\left( \lambda \right) = \frac{{\partial \lambda_{0} }}{{\partial n_{analyte} }} \simeq \frac{{{\Delta }\lambda_{0} }}{\delta n} {\text{(nm}}/{\text{RIU)}}$$

where nm/RIU is the sensitivity (${\text{S}}$) unit. In this regard, FoM is another sensing parameter to investigate the resolution of the optical RI sensor. FoM is calculated by normalizing the sensitivity (${\text{S}}$) to the bandwidth of the full width at half the maximum (FWHM) at the resonance mode. FoM is calculated as follows^53^:

4 $${\text{FoM}} = \frac{{\text{S}}}{{{\text{FWHM}}}} {\text{(RIU}}^{{ - 1}} {)}$$

By increasing sensitivity and decreasing FWHM bandwidth, FoM is increased. FoM is also proportional to the quality factor ($Q$), where $Q = \lambda_{0} /{\text{FWHM}}$. Therefore, FoM can be expressed as ${\text{FoM}} = {\text{S}}.Q/\lambda_{0}$. As a result, for having a desirable optical RI sensor, high sensitivity and high FoM are required.

## Theory and analysis of surface plasmon excitation in PhC structures

In order for photons to be able to excite surface plasmons, they must have the same frequencies and momentums. The dispersion relation for an SPP propagating at the metal–dielectric interface can be derived as:

5 $$k_{SPP} = \frac{\omega }{c}\sqrt {\frac{{\varepsilon_{m} \varepsilon_{d} }}{{\varepsilon_{m} + \varepsilon_{d} }}}$$

where $\omega$ is the frequency of incident light, and $c$ is the speed of light in a vacuum. $\varepsilon_{m}$ and $\varepsilon_{d}$ are frequency-dependent permittivity of metal and dielectric (here it is analyte), respectively. The wave vector (k) and the dispersion curve provide us with a better comprehension of the properties of the propagation modes and the coupling necessities for wave vector matching.

LSPs are localized plasma oscillations that can be excited on the surfaces of metallic nanoparticles. Excitation of LSPs causes the electromagnetic fields near the particle's surface are greatly enhanced. In contrast to propagating surface plasmons, LSPs do not require special lighting arrangement for phase matching and they can be excited easily by direct irradiation of light^63,64^.

Knowing that the momentum of a photon propagating in free space ($k_{0} = \omega /c$) is less than the momentum of surface plasmons, they do not have the same momentum at any given frequency. Thus surface plasmons at the metal–dielectric interface cannot be excited directly by optical beams^65^. Hence, to excite propagating surface plasmons in plasmonic devices, one has to use coupling techniques such as prisms, gratings, fibres, and waveguides^50^. In this regard, surface plasmons induced by a plasmonic element can be excited by a PhC waveguide mode. The linear dispersion relation of a PhC waveguide can be approximated as follows^66^:

6 $$\omega_{k} = \frac{c}{{n_{eff} }}\left( {\frac{\pi }{a} - \sqrt {D^{2} + \left( {k - \frac{\pi }{a}} \right)^{2} } } \right)$$

where $a$ is the lattice constant, $D$ is the size of the photonic band gap (PBG), and $n_{eff}$ is the effective index of refraction. Given that the effective refractive index of a PhC structure is greater than the refractive index of air (n = 1), thereby the wave vector ($k$) of a PhC structure is larger than that of air (free space). Therefore, for the proposed hybrid P-PhC structure, the momentum of surface plasmons propagating at the metal–dielectric interface can be matched with the momentum of PhC modes in the PBG region. Therefore, the coupled LSPs in the PhC structure can be excited by PhC modes without the requirement of other momentum-matching techniques. The excitation of surface plasmons in the PhC depends on the position and geometric parameters of the metal elements.

## The proposed hybrid P-PhC sensor's configuration

**Figure 2:**
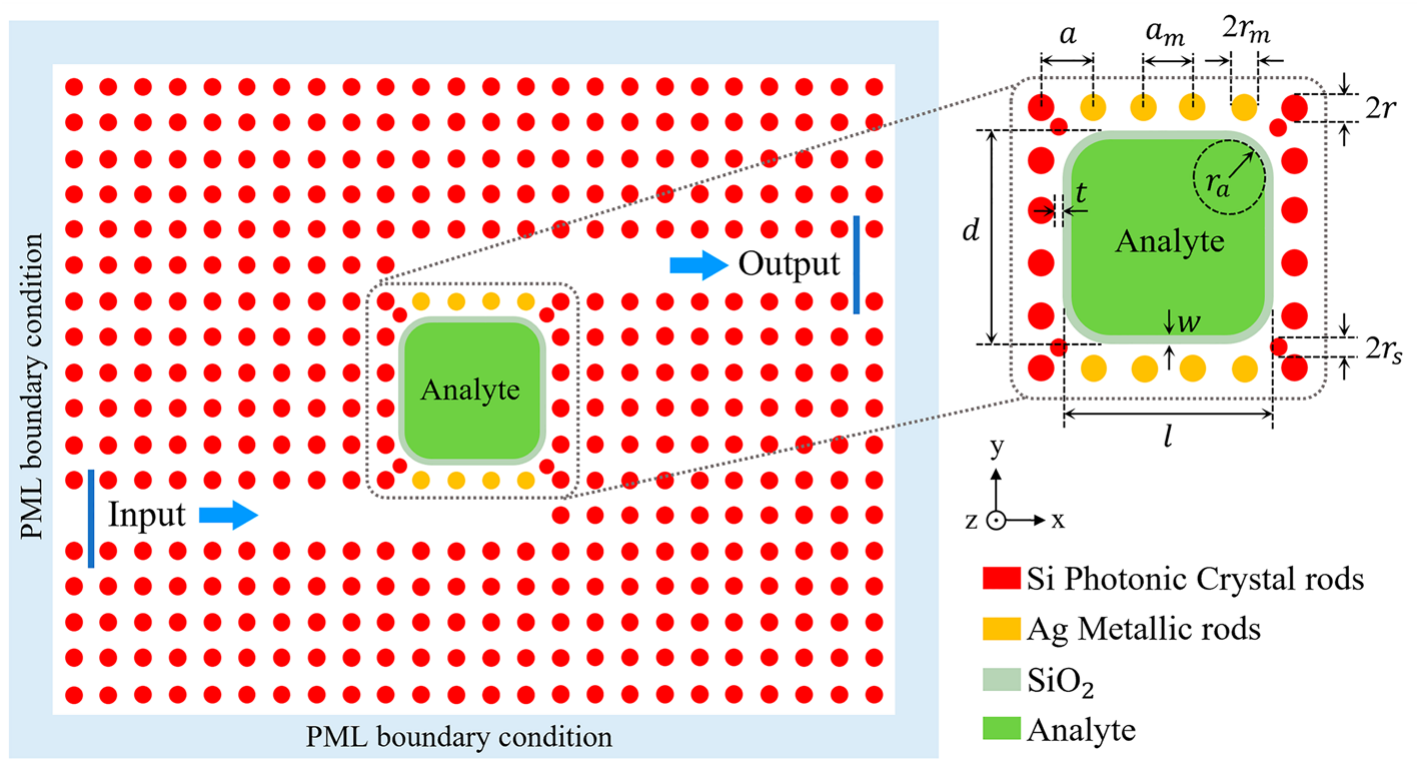
2D schematic view of the proposed hybrid P-PhC RI sensor structure.

The two-dimensional (2D) schematic view of the proposed hybrid P-PhC RI sensor structure is illustrated in Fig. 2. The structure consists of a rod-type PhC structure and some metallic rods, which are located in the coupling regions between waveguides and the resonant cavity of PhC. In this structure, a 2D square lattice of silicon (Si) rods surrounded by an air background is used. Their RIs are n = 3.45 and n = 1, respectively. The lattice constant is assumed to be $a$ = 645 nm. The radii of Si rods is $r$ = 0.22 $a$. As illustrated in Fig. 2, the PhC structure has a resonant cavity, an input waveguide at the bottom side of the cavity, and an output waveguide at the top side of the cavity. At the corners of the resonant cavity, four Si scatterer rods with the reduced radius of *r*_*s*_ = 0.18*a* are used to prevent light dispersion and backscattering into the waveguides.

As seen in Fig. 2, For the proposed structure, the resonant cavity is subjected to the analyte surrounded by a thin SiO_2_ layer (n = 1.42). When the electromagnetic field overlaps with the analyte area, the RI analyte variations, resulting in a shift in the resonance wavelength. To enhance field overlapping with the analyte, some metallic rods are employed at the top and bottom sides of the resonant cavity at the coupling regions between input and output waveguides and the cavity. The lattice constant of metallic rods is assumed to be *a*_*m *_= *a*. The radii of metallic rods are assumed to be equivalent to the radii of Si PhC rods ($r_{m}$ = $r$ = 0.22$a$). The material chosen for metallic rods is silver (Ag), due to its much lower absorption compared to other metals. Here, the permittivity function of Ag is modeled based on the experimental Johnson and Christy results^67^. To prevent the oxidation of Ag metal, a dielectric layer of SiO_2_ is located between the analyte solution and the Ag metallic rods. Thereby, the whole parts of the sensor structure will not be exposed to the analyte. All of the structural parameters of the proposed sensor structure are summarized in Table 1.

**Table 1:** Structural parameters of the proposed hybrid P-PhC RI sensor structure.

Parameter	Symbol	Quantity	Unit
Lattice constant of Si rods	*a*	645	nm
Lattice constant of metallic rods	*a*_*m*_	645	nm
Si PhC rods radius	$r$	0.22 $a$	–
Si scatterer rods radius	$r_{s}$	0.18 $a$	–
Ag metallic rods radius	$r_{m}$	0.22 $a$	–
SiO_2_ layer width	$w$	20	nm
SiO_2_ layer curvature radius in corners	$r_{a}$	650	nm
Distance between rods and SiO_2_ layer	$t$	40	nm
Analyte region length	$l$	2.85	μm
Analyte region width	$d$	2.85	μm

In the proposed sensor structure, a Gaussian source light is launched into the input waveguide of PhC, which supports the transverse magnetic (TM) polarization. The surface plasmons induced by the metallic rods on the bottom side of the resonant cavity are excited by the coupling of incoming light from the input waveguide into the cavity. Then, the surface plasmons induced by the metallic rods on the top side of the resonant cavity are excited by coupling light from the cavity towards the output waveguide. In this hybrid structure, the proper excitation of metal-induced surface plasmons is very important. The excited LSPs are cooperatively coupled to the PhC guiding modes to generate a hybrid PhC-P mode.

**Figure 3:**
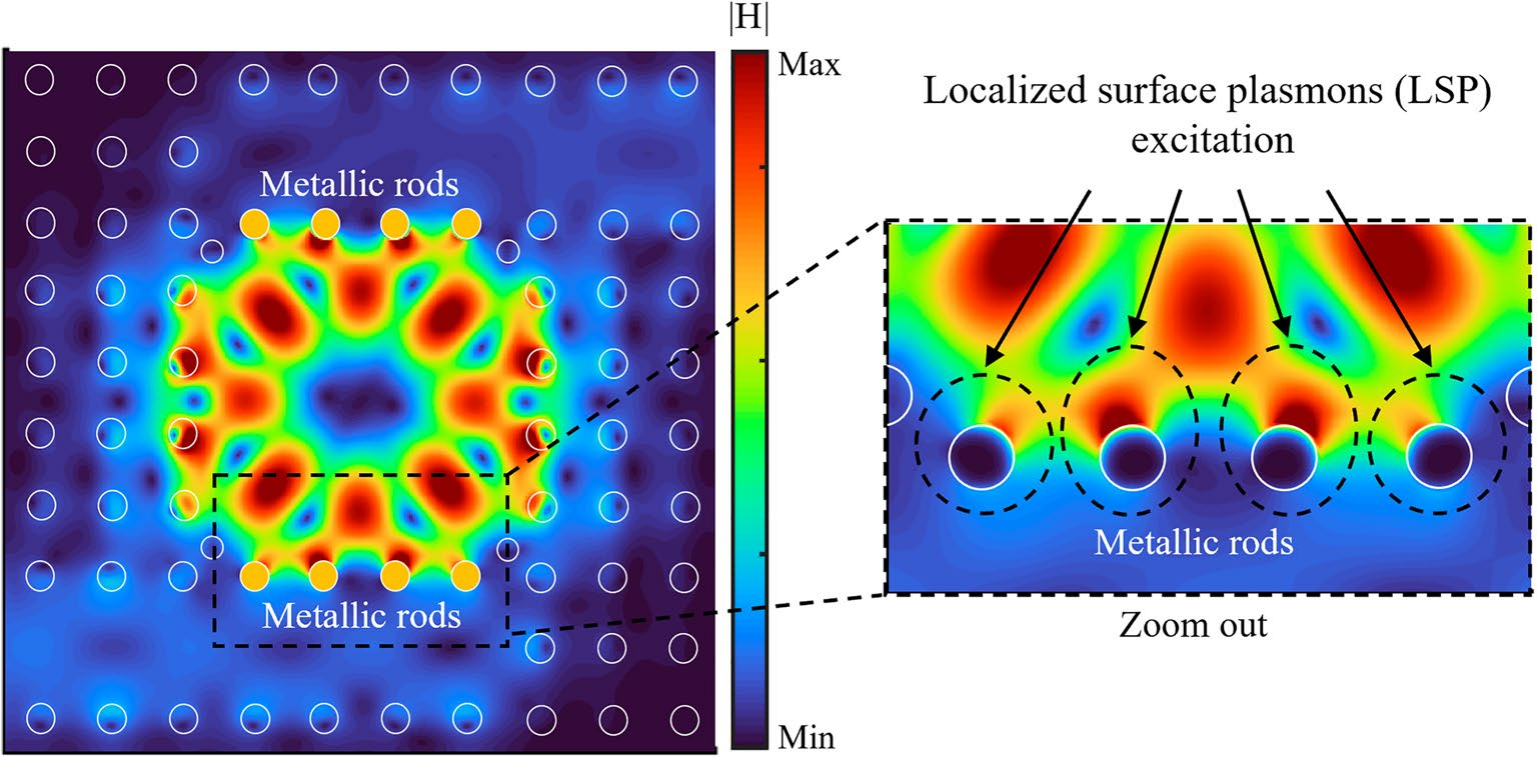
The magnetic field distribution (|H|) for the proposed hybrid P-PhC sensor at the resonance wavelength of 1860 nm.

To gain a better insight, Fig. 3 illustrates the magnetic field distribution (|H|) for the proposed hybrid sensor at the resonance wavelength of 1860 nm. In this figure, the metallic rods are schematically marked using yellow circles. But these yellow circles are removed in the zoomed figure to see the partial penetration of the field into the metallic rods. It can be seen that the LSPs induced by metallic rods in coupling regions are properly excited in the PhC structure. As seen in the zoomed section in Fig. 3, the optical field is strongly confined at the metal–dielectric interface of metallic rods and extended toward the analyte area in the cavity. Whereas for the Si rods, most of the optical field has penetrated inside rods. Therefore, by combining the LSPs of the metallic rods with PhC guiding modes, a hybrid PhC-P mode is generated to increase the sensitivity and resolution of the proposed sensor.

**Figure 4:**
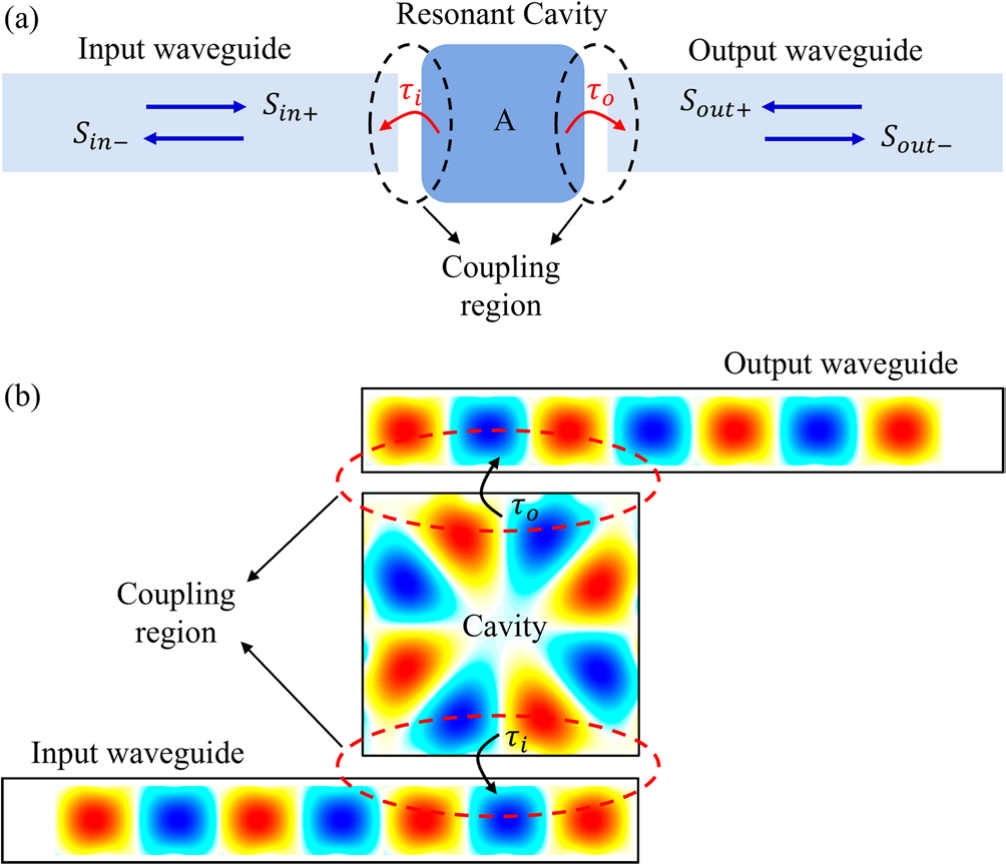
(**a**) Schematic of a coupled waveguide-cavity-waveguide system for the coupled-mode theory. (**b**) Schematic of the light coupling mechanism for the proposed hybrid P-PhC RI sensor structure.

In order to describe the mechanism behind guiding light from the input waveguide to the output waveguide, the coupled-mode theory can be used. The structure shown in Fig. 4a is a well-known schematic model based on coupled-mode theory for describing optical filters based on waveguides end-coupled to a resonator. In this structure, a resonant cavity is connected to two single-mode waveguides which form the input and output ports. Light is coupled from the input waveguide to the resonant cavity, then is coupled to the output waveguide. In the resonant cavity, there is a resonant mode with the frequency of $\omega_{0}$ which decays with lifetimes of $\tau_{i}$ and $\tau_{o}$ into the two waveguides. Subsequently, a schematic of the light coupling system for the proposed sensor structure is shown in Fig. 4b. The structure of Fig. 4b is a special case of the structure of Fig. 4a. In the proposed sensor structure, the electric field profiles in the resonant cavity and the waveguides are obtained separately and placed together schematically in Fig. 4b to provide a better prospect. In reality the field intensity is much higher in the resonant cavity. It is worth mentioning that the resonance in our case is of a standing wave nature and unlike ring resonators, light does not rotate inside the cavity. Since the resonance profile of the cavity is similar to the waveguide mode profile it can act as a feed and excite the guided modes in the waveguides. Although the coupling regions seem similar to directional couplers, but the coupling mechanism is not the same since light does not rotate inside the cavity. Similar to the structure of Fig. 4a, the lifetimes of $\tau_{i}$ and $\tau_{o}$ are also applicable for the coupling regions in Fig. 4b.

In Fig. 4a, $S_{in + }$ and $S_{in - }$ are the input/output field amplitudes of the mode in the input waveguide, and $S_{out + }$ and $S_{out - }$ are the input/output field amplitudes of the mode in the output waveguide. The field amplitude of a single resonant mode in the cavity is $A$. Considering $S_{out + } = 0$, the transmission spectrum of a coupled waveguide-cavity-waveguide system can be theoretically defined using the coupled-mode theory as follows^68^:

7 $$- i\omega A = - i\omega_{0} A - \frac{A}{{\tau_{i} }} - \frac{A}{{\tau_{o} }} + \sqrt {\frac{2}{{\tau_{i} }}} S_{in + }$$

8 $$S_{in - } = - S_{in + } + \sqrt {\frac{2}{{\tau_{i} }}} A$$

9 $$S_{out - } = \sqrt {\frac{2}{{\tau_{o} }}} A$$

The transmission spectrum can be obtained as follows^68^:

10 $$T\left( \omega \right) = \frac{{\left| {S_{out - } } \right|^{2} }}{{\left| {S_{in + } } \right|^{2} }} = \frac{{\frac{2}{{\tau_{o} }}\left| A \right|^{2} }}{{\left| {S_{in + } } \right|^{2} }} = \frac{{\frac{4}{{\tau_{i} \tau_{o} }}}}{{\left( {\omega - \omega_{0} } \right)^{2} + \left( {\frac{1}{{\tau_{i} }} + \frac{1}{{\tau_{o} }}} \right)^{2} }}$$

Assuming symmetry condition $\tau_{i} = \tau_{o}$, the total lifetime is given by $1/\tau = 1/\tau_{i} + 1/\tau_{o} = 2/\tau_{i}$. According to $Q = \omega_{0} \tau /2$ and $1/\tau_{i} = 1/\tau_{o} = \omega_{0} /4Q$, the transmission spectrum in terms of quality factor $Q$ can be expressed as follows^68^:

11 $$T\left( \omega \right) = \frac{{\frac{1}{{4Q^{2} }}}}{{\left( {\frac{{\omega - \omega_{0} }}{{\omega_{0} }}} \right)^{2} + \frac{1}{{4Q^{2} }}}}$$

**Figure 5:**
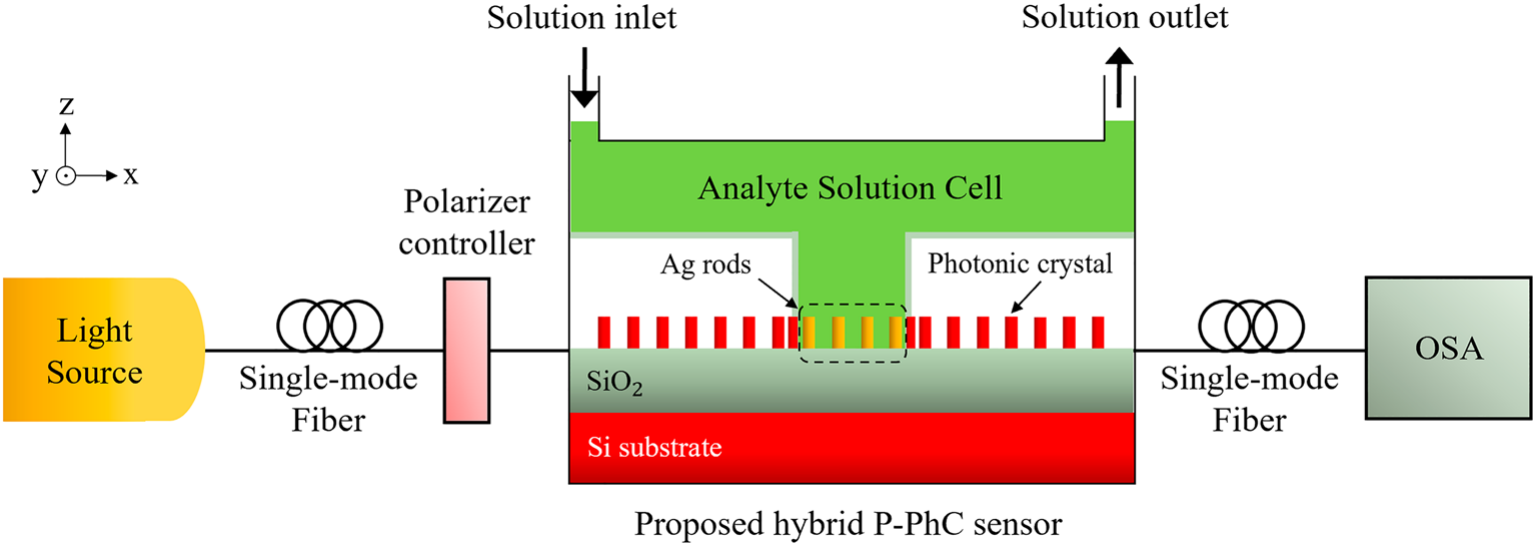
The schematic of the experimental setup used for the proposed hybrid P-PhC RI sensor.

Figure 5 illustrates a schematic view of the experimental setup used for the proposed hybrid P-PhC RI sensor. This setup includes an optical light source, polarization controller, optical spectrum analyzer (OSA), computer, and the proposed hybrid P-PhC RI sensor. As seen, for the proposed hybrid sensor structure, a microfluidic channel is integrated at the center of the sensing analyte region for improvement of the stability and accuracy of the sensor. The analyte solution enters the analyte microfluidic cell through the solution inlet and exits through the solution outlet.

### Ethical approval

We the undersigned declare that the manuscript entitled “Hybridization of Surface Plasmon Polaritons and Photonic Crystal Resonators for High-Sensitivity and High-Resolution Sensing Applications” is original, has not been fully or partly published before, and is not currently being considered for publication elsewhere. Also, results are presented clearly, honestly, and without fabrication, falsification, or inappropriate data manipulation. We confirm that the manuscript has been read and approved by all named authors and that there are no other persons who satisfied the criteria for authorship but are not listed. We further confirm that the order of authors listed in the manuscript has been approved by all of us.

## Results and discussions

**Figure 6:**
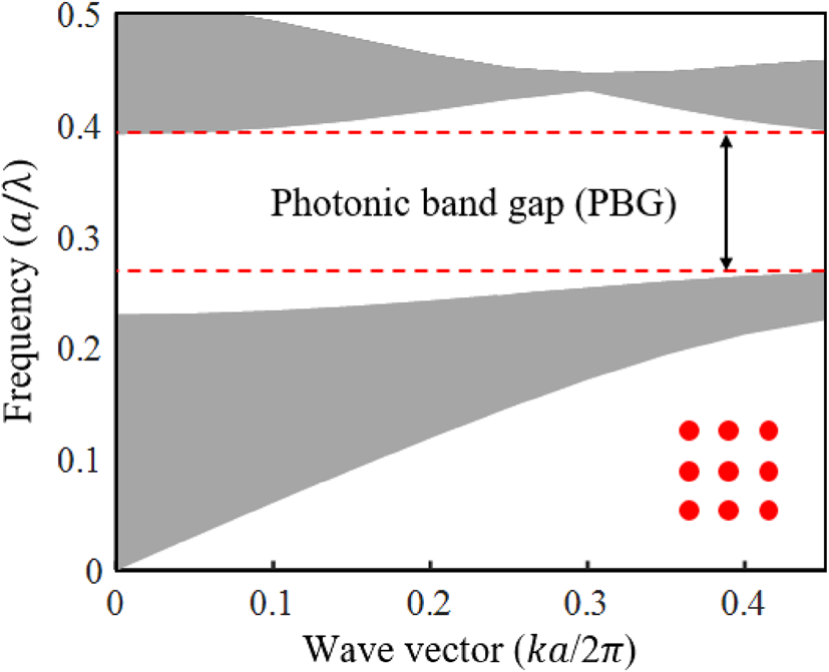
The band structure of PhC used in the proposed hybrid P-PhC RI sensor.

The finite-difference time-domain (FDTD) method is utilized to evaluate the sensing performance of the proposed hybrid sensor structure and the effects of the metallic rods in the hybrid sensor structure. Perfectly matched layers (PML) boundary conditions are used in the x- and y-directions to absorb the waves emitted outside of the structure. In all directions, the sensor structure is meshed by the size of 5 nm. The band structure of PhC used in the proposed hybrid PhC-P structure can be calculated using the plane wave expansion (PWE) method. Figure 6 depicts the PBG range in the band structure of PhC used in the proposed hybrid P-PhC RI sensor. The calculated PBG has a normalized frequency range of 0.28–0.38 ($a/\lambda$). Thus, the wavelength range is equal to 1666 nm to 2307 nm.

**Figure 7:**
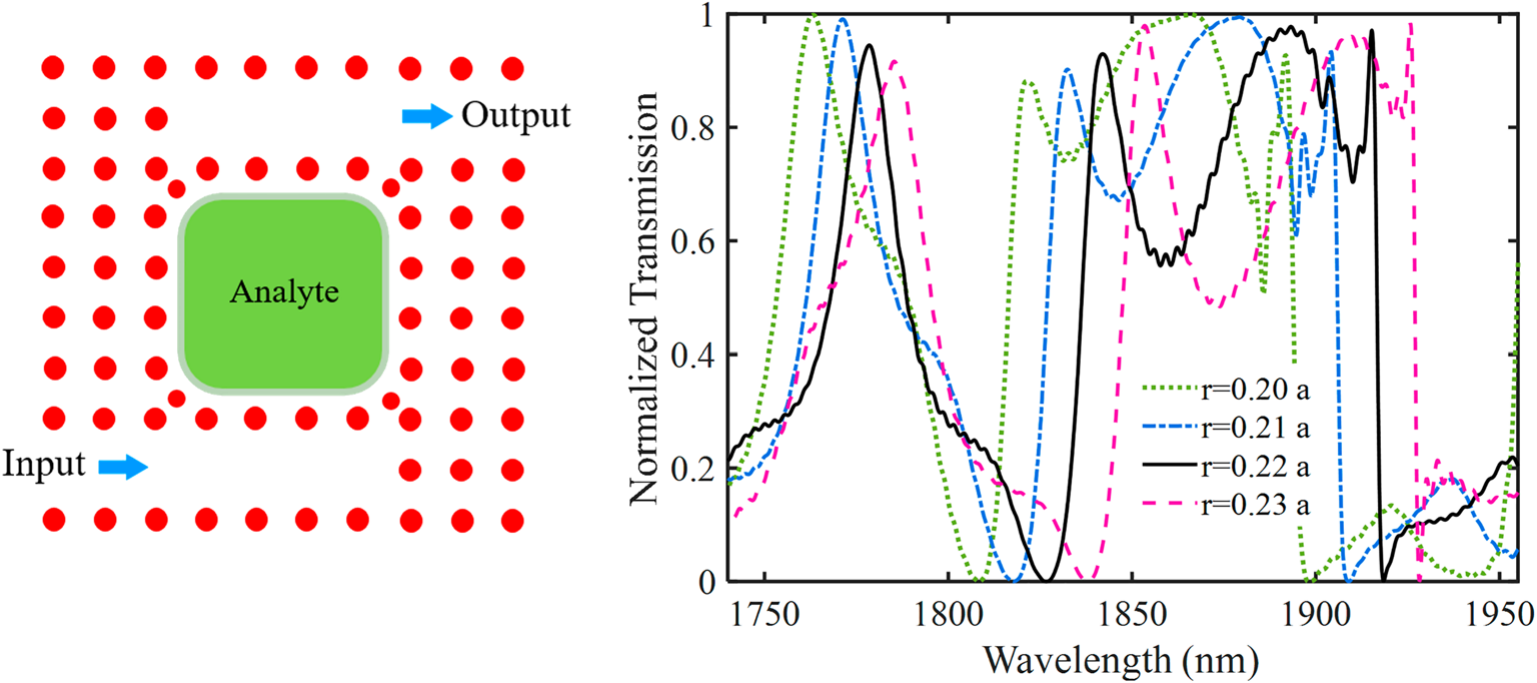
The effect of the radius of Si PhC rods ($r$) on the transmission spectrum of the PhC sensor without metallic rods.

**Figure 8:**
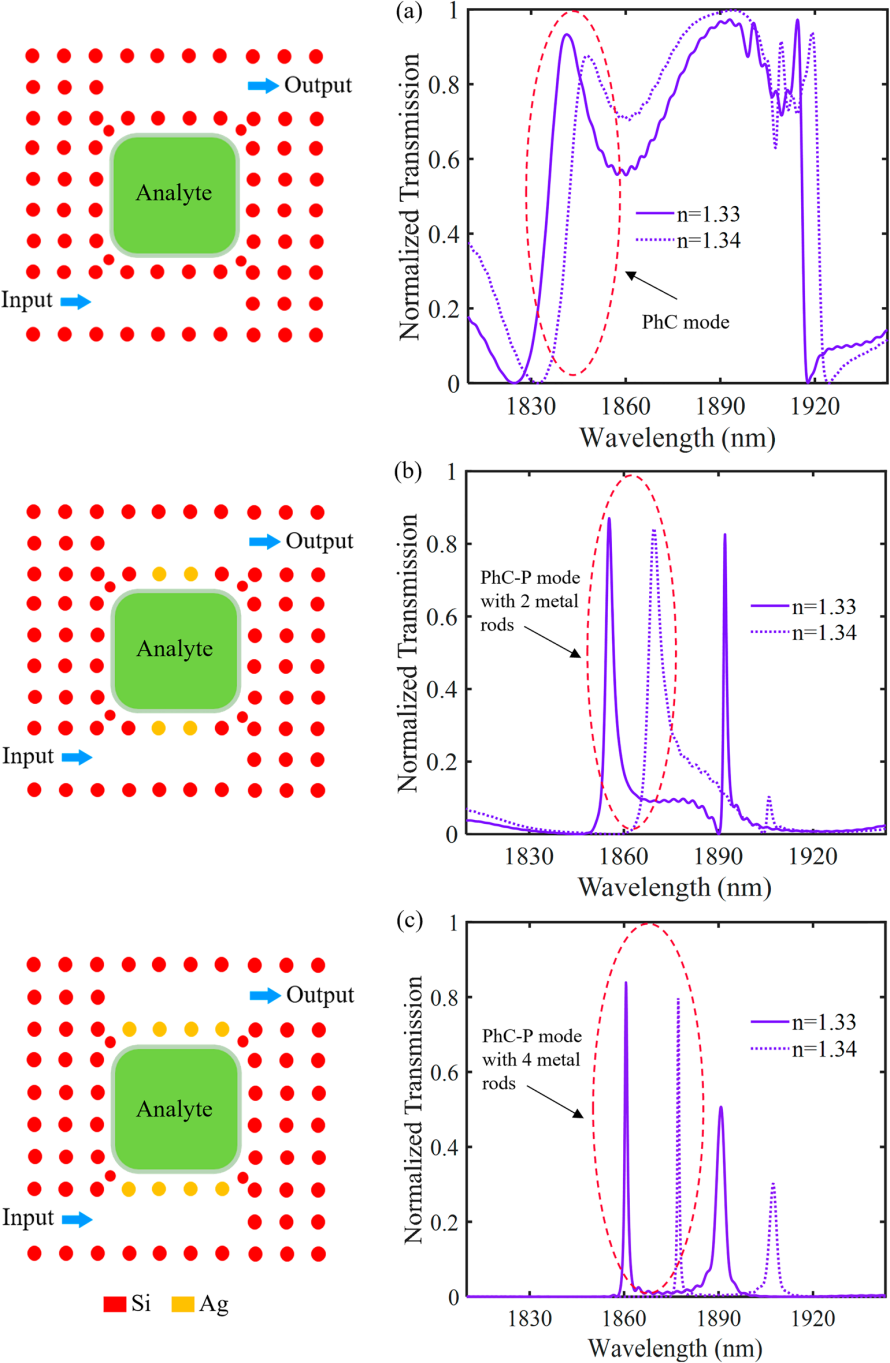
The effect of analyte RI variation on the transmission spectrums of three RI sensor structures including (**a**) PhC sensor without metallic rods, (**b**) hybrid P-PhC sensor with two metallic rods, and (**c**) proposed hybrid P-PhC sensor with four metallic rods in coupling regions.

Figure 7 illustrates the effect of the radius of Si PhC rods ($r$) on the transmission spectrum of the PhC sensor without metallic rods. The analyte base is set to be water (n = 1.33). As seen, the transmission spectrum of the PhC sensor has multi resonance modes. Here, we have considered a resonance at the wavelength of 1855 nm. By increasing the $r$ size, the resonance wavelength is shifted toward higher wavelengths. We have selected $r$ = 0.22 $a$. To further evaluate the sensing performance of the proposed sensor, the effect of analyte RI variation on the transmission spectrums of three RI sensor structures including a PhC sensor without metallic rods, a hybrid P-PhC sensor with two metallic rods in coupling regions, and the proposed hybrid P-PhC sensor with four metallic rods in coupling regions are illustrated in Fig. 8a–c. It is assumed that the analyte RI is changed from 1.33 to 1.34.

As shown in Fig. 8a, the transmission spectrum of the PhC sensor structure without metallic rods has some resonance modes. Here, for comparison of three structures, we have considered the resonance mode at the wavelength of 1855 nm with an FWHM bandwidth of about 20 nm. As seen, by changing the analyte RI from 1.33 to 1.34, the resonance wavelength shifts by 6.55 nm. It provides a sensitivity value of 655 nm/RIU and an FoM value of 33 RIU^−1^.

Figure 8b illustrates the hybrid P-PhC sensor with two metallic rods in coupling regions. It has two resonance modes. We have considered the resonance mode at the wavelength of 1856 nm with a bandwidth of about 2.7 nm. It can be seen that adding metallic rods instead of Si rods in coupling regions reduces the FWHM bandwidth. Although, these metallic rods have ohmic losses. From this figure, for the analyte RI changing from 1.33 to 1.34, a 14.07 nm wavelength shift is observed. It leads to a high sensitivity value of 1407 nm/RIU and a good FoM value of 521 RIU^−1^. The sensitivity and FoM values of the hybrid P-PhC sensor with two metallic rods are about 2.14 and 15.7 times larger than that of the purely PhC sensor structure, respectively. Figure 8c illustrates the hybrid P-PhC sensor with four metallic rods in coupling regions. We have considered the resonance mode at the wavelength of 1860 nm. It has an ultra-narrow FWHM bandwidth of 0.75 nm. From this figure, by changing the analyte RI from 1.33 to 1.34, the resonance wavelength experiences a considerable shift by 16.38 nm. A higher sensitivity value of 1638 nm/RIU and a larger FoM value of 2184 RIU^−1^ are obtained. These obtained values are about 1.16 and 4.19 times larger than that of the hybrid P-PhC sensor with two metallic rods, respectively. Finally, these values are about 2.5 and 66.18 times larger than that of the pure PhC sensor, respectively. According to the obtained results, the proposed hybrid structure can act as a high-performance RI sensor for bio-sensing applications.

**Figure 9:**
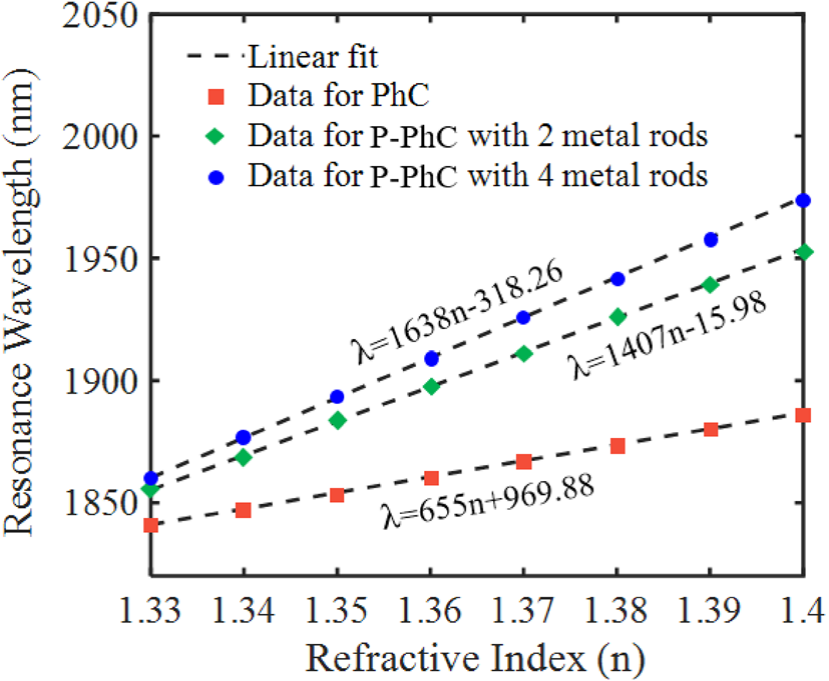
Resonance wavelength of three sensor structures as a function of analyte refractive indices.

**Figure 10:**
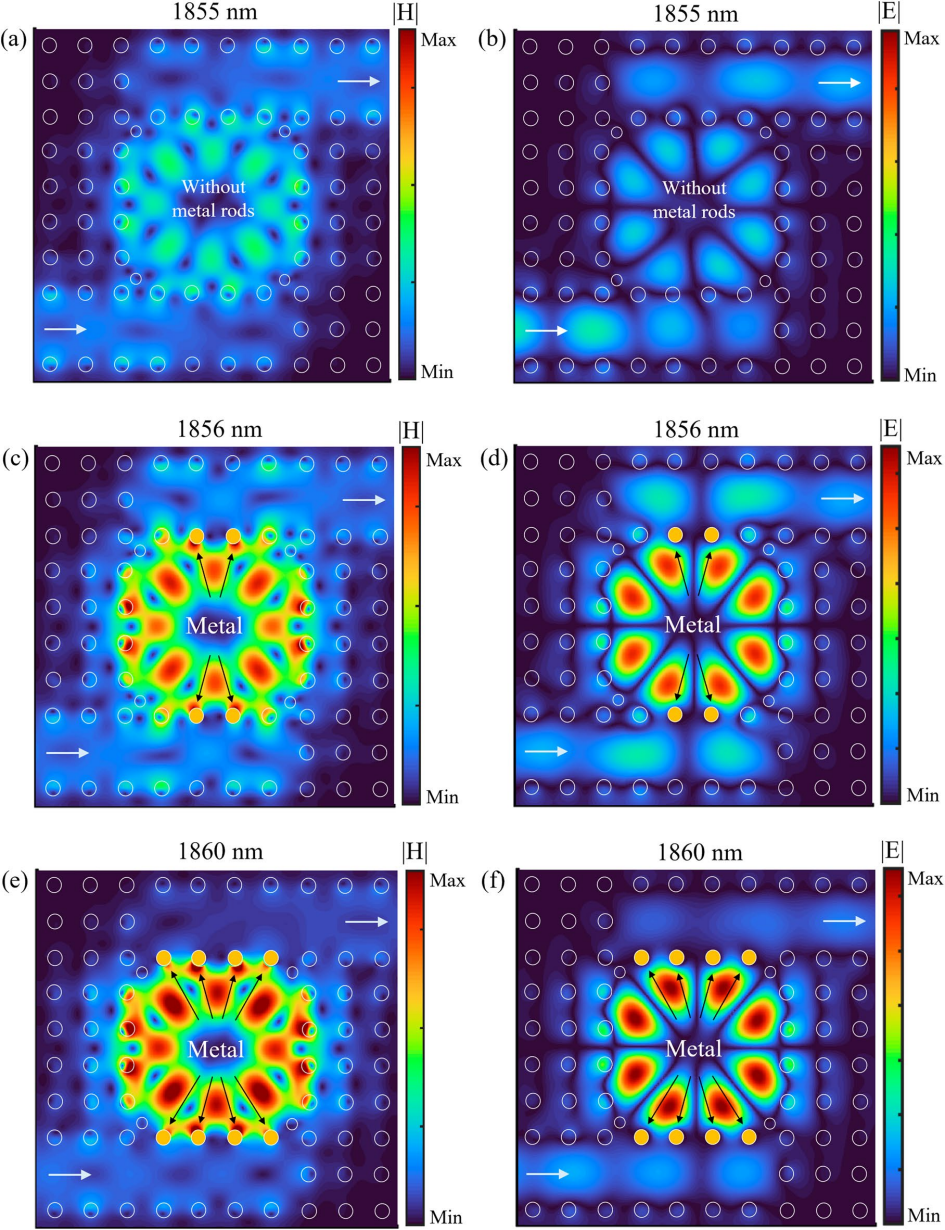
The distributions of magnetic and electric fields (|H| and |E|) for three RI sensor structures including (**a**) |H| for PhC sensor without metallic rods at 1855 nm, (**b**) |E| for PhC sensor without metallic rods at 1855 nm (**c**) |H| for hybrid P-PhC sensor with two metallic rods at 1856 nm, (**d**) |E| for hybrid P-PhC sensor with two metallic rods at 1856 nm, (**e**) |H| for the proposed hybrid P-PhC sensor with four metallic rods at 1860 nm, and (**f**) |E| for the proposed hybrid P-PhC sensor with four metallic rods at 1860 nm.

Figure 9 illustrates a linear relationship between the considered resonance wavelength and the refractive indices from n = 1.33 to 1.4 for the three sensor structures (a PhC sensor without metallic rods, a hybrid P-PhC sensor with two metallic rods, and the proposed hybrid P-PhC sensor with four metallic rods). In this figure, the calculated slopes of the linear curves estimate the sensitivity value of the RI sensors. The sensitivity of the proposed hybrid P-PhC sensor with four metallic rods is a higher value in comparison with the other two sensor structures. To be able to have a better insight, Fig. 10a–c illustrates the distributions of magnetic and electric fields (|H| and |E|) at the considered resonance modes for three sensor structures, respectively. Figure 10a,b illustrate the field profiles of |H| and |E| for the pure PhC sensor at the resonance wavelength of 1855 nm, respectively. It is observed that maximum field distribution is mainly concentrated within the resonant cavity.

Figure 10c,d illustrate the field profiles of |H| and |E| for the hybrid P-PhC sensor with two metallic rods at the resonance wavelength of 1856 nm, respectively. As seen, the field is strongly confined at the metal–dielectric interface. It confirms the excitation of LSPs induced by metallic rods. Compared to the purely PhC sensors, the field intensity for the hybrid P-PhC sensor with two metallic rods is stronger. This represents a larger volume of interaction between the analyte area and the optical field. Enhancing the interaction volume provides a larger sensitivity to RI variations. Figure 10e,f illustrate the field profiles of |H| and |E| for the hybrid P-PhC sensor with four metallic rods at the resonance wavelength of 1860 nm, respectively. As seen, the field intensity at the metal–dielectric interface and within the cavity is much stronger as compared to the purely PhC sensor and the hybrid P-PhC sensor with two metallic rods.

**Figure 11:**
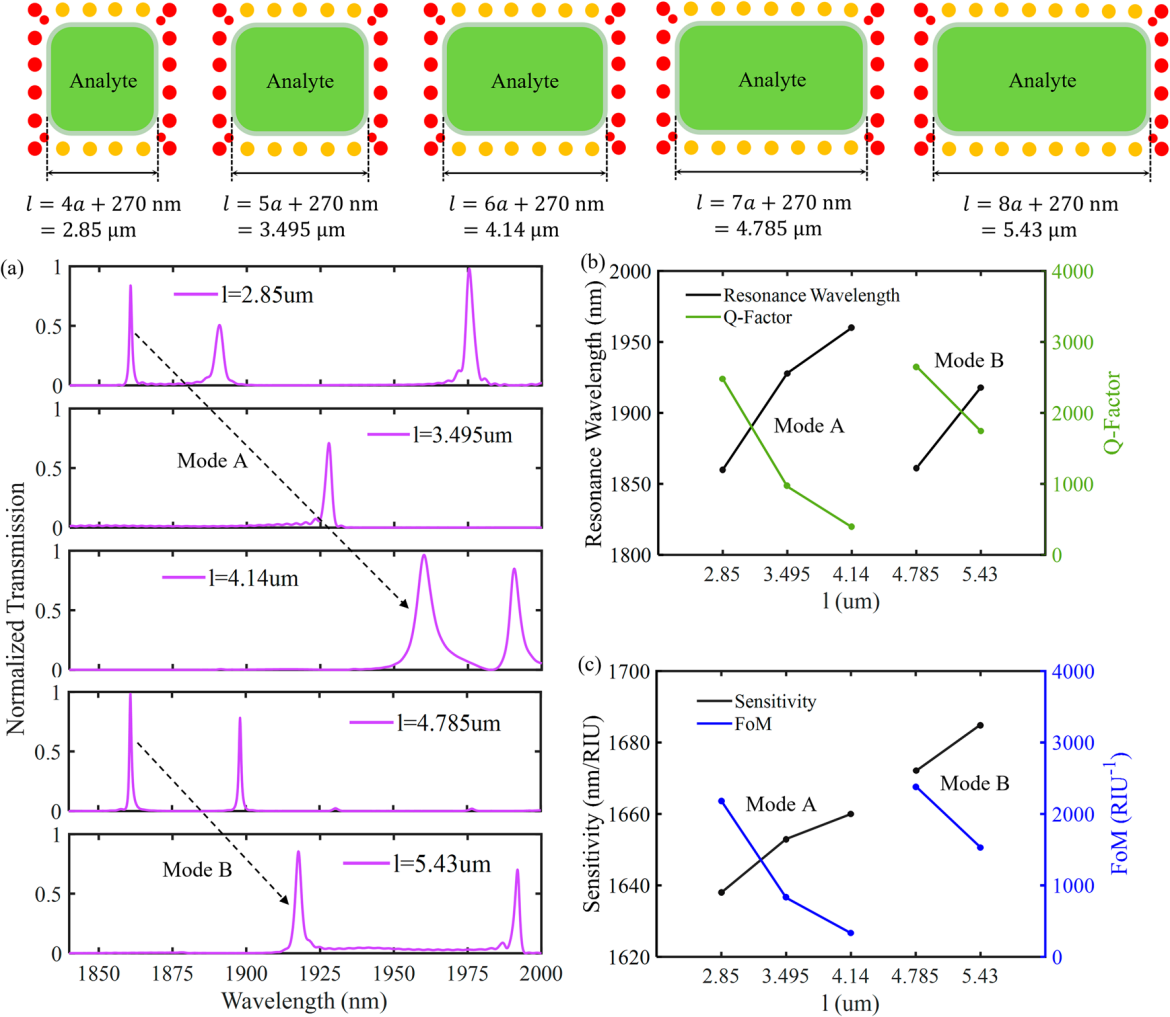
(**a**) The normalized transmission spectrum as a function of the analyte region length ($l$), (**b**) The effect of the length on the resonance wavelength and quality factor, (**c**) The effect of the length on the sensitivity and FoM.

Figure 11a illustrates the effect of the cavity length ($l$) variations on the normalized transmission spectrum of the proposed hybrid P-PhC sensor. The length is increased from 2.85 to 5.43 μm by a 645 μm step. The other geometric parameters are kept unchanged. As shown in this figure, the resonance mode obtained for $l$ = 2.85 μm, $l$ = 3.495 μm and $l$ = 4.14 μm is marked by “Mode A”, and the resonance mode obtained for $l$ = 4.785 μm and $l$ = 5.43 μm is marked by “Mode B”. Figure 11b shows that by incrementing the length, modes A and B have a redshift towards the higher wavelengths, and quality factor is decreased. When the analyte region length is $l$ = 4.785 μm, its resonance behavior at the wavelength of 1861 nm is equivalent to the case where two analyte regions with a length of 2.85 μm are connected to each other. The effect of the change of length is evaluated on sensing performance in Fig. 11c. It is expected that the sensor sensitivity be increased with the enlargement of the sensing area. However, increasing cavity size will decrease the quality factor and enhance loss. This is due to the fact that the coupling coefficient will also be increased which reduces the quality factor. Based on Fig. 11c, by increasing the length, the sensitivity is enhanced and FoM is decreased. The maximum sensitivity of 1685 nm/RIU is obtained for $l$ = 5.43 $\mu$ m, and the maximum FoM of 2388 RIU^−1^ is seen for $l$ = 4.785 μm. Therefore, we selected $l$ = 4.785 μm for the proposed hybrid P-PhC sensor structure.

**Figure 12:**
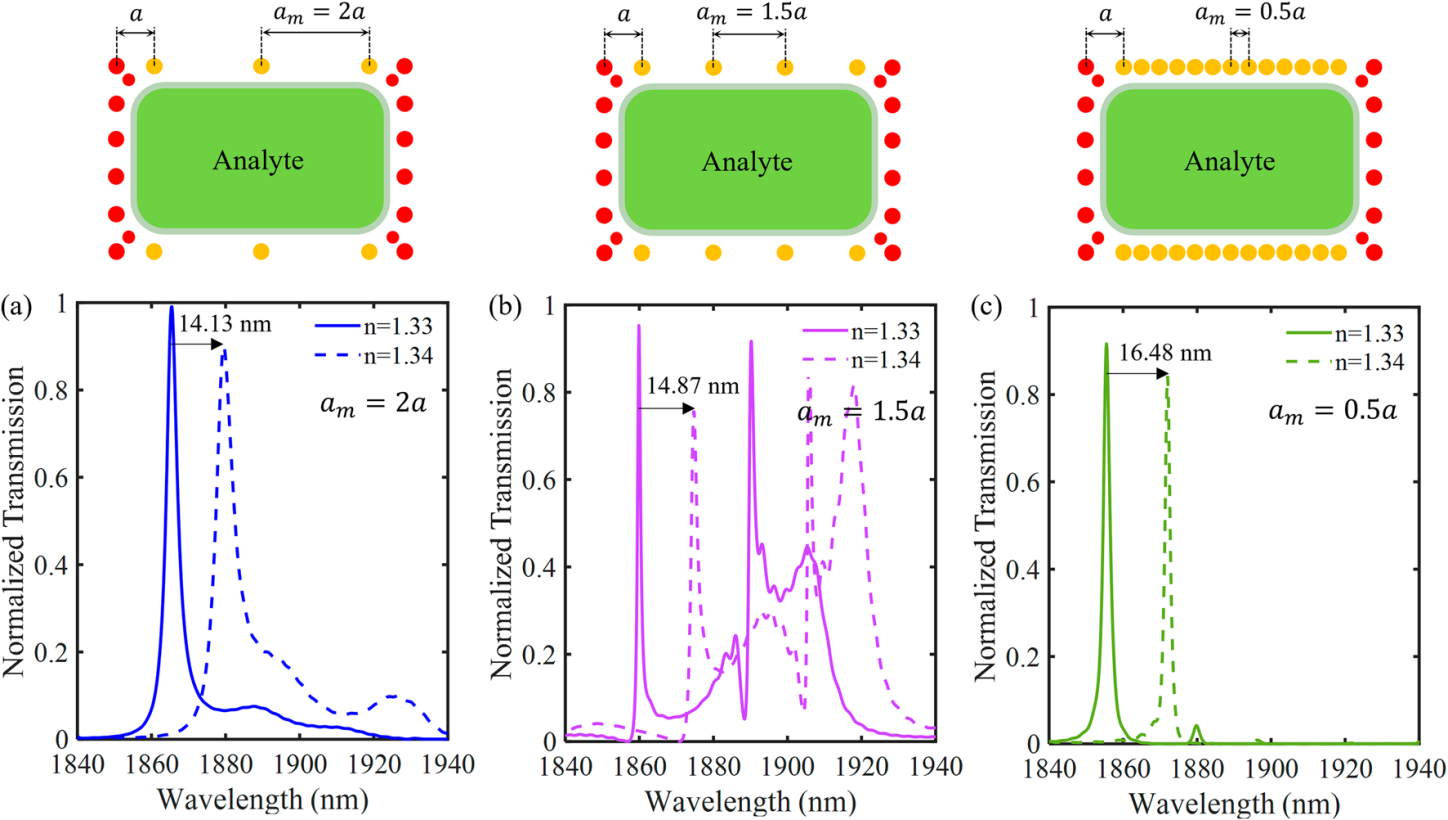
The normalized transmission spectrum as a function of the period of metallic rods for (**a**) $a_{m}$ = 2$a$, (**b**) $a_{m}$ = 1.5$a$ and (**c**) $a_{m}$ = 0.5$a$.

Figure 12 illustrates the effect of changing the period of metallic rods on the normalized transmission spectrum of the proposed hybrid P-PhC sensor, for which $l$ = 4.785 μm. The lattice constant of metallic rods is marked by “$a_{m}$”. Figure 12a–c show cases where $a_{m}$ = 2 $a$, $a_{m}$ = 1.5 $a$ and $a_{m}$ = 0.5 $a$, respectively. The other geometric parameters are kept unchanged. As shown in Fig. 12a, by changing the analyte RI from 1.33 to 1.34, the resonance wavelength is shifted by 14.13 nm. Thus, a sensitivity of 1413 nm/RIU is obtained for $a_{m}$ = 2 $a$. Subsequently, Fig. 12b,c show a sensitivity of 1487 nm/RIU and 1648 nm/RIU for $a_{m}$ = 1.5 $a$ and $a_{m}$ = 0.5 $a$, respectively. Moreover, the calculated quality factors for $a_{m}$ = 2 $a$, $a_{m}$ = 1.5 $a$ and $a_{m}$ = 0.5 $a$ are 622, 2657 and 928, respectively. As the field leakage is increased, the lifetime $\tau$ is decreased. As a result, by increasing the period of metallic rods $Q$ is reduced. On the other hand, by decreasing the period, the number of metallic rods is incremented. This increases the absorption of the field by the metallic rods. Therefore, $Q$ is decreased. Hence, we selected $a_{m}$ = $a$.

**Figure 13:**
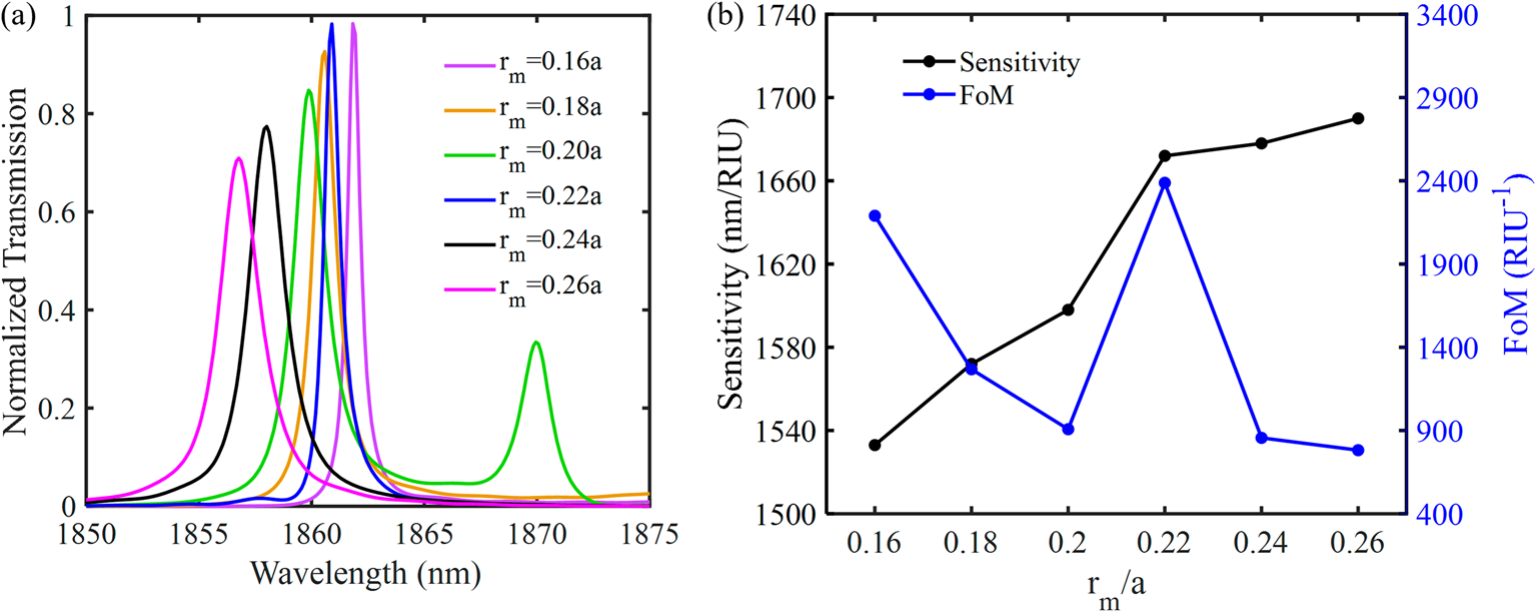
(**a**) The normalized transmission spectrum as a function of the radius of metallic rods ($r_{m}$), (**b**) The effect of the radius of the metallic rods on the sensitivity and FoM.

Figure 13a illustrates the effect of the radius of the metallic rods ($r_{m}$) variations on the normalized transmission spectrum of the proposed hybrid P-PhC sensor, for which $l$ = 4.785 $\mu$ m. The radius of the metallic rods is increased from 0.16 to 0.26$a$ by a 0.02$a$ step. The other geometric parameters are kept unchanged. As seen in Fig. 13b, when $r_{m}$ is increased, the sensitivity is enhanced, FoM is also decreased and it has the maximum value only when $r_{m} = r$. This is due to the fact that by decreasing the radius, the field leakage in the coupling region is increased. On the other hand, by increasing the radius of the metallic rods, the field absorption by the metallic rods is increased. Hence, we selected $r_{m} = r$.

**Figure 14:**
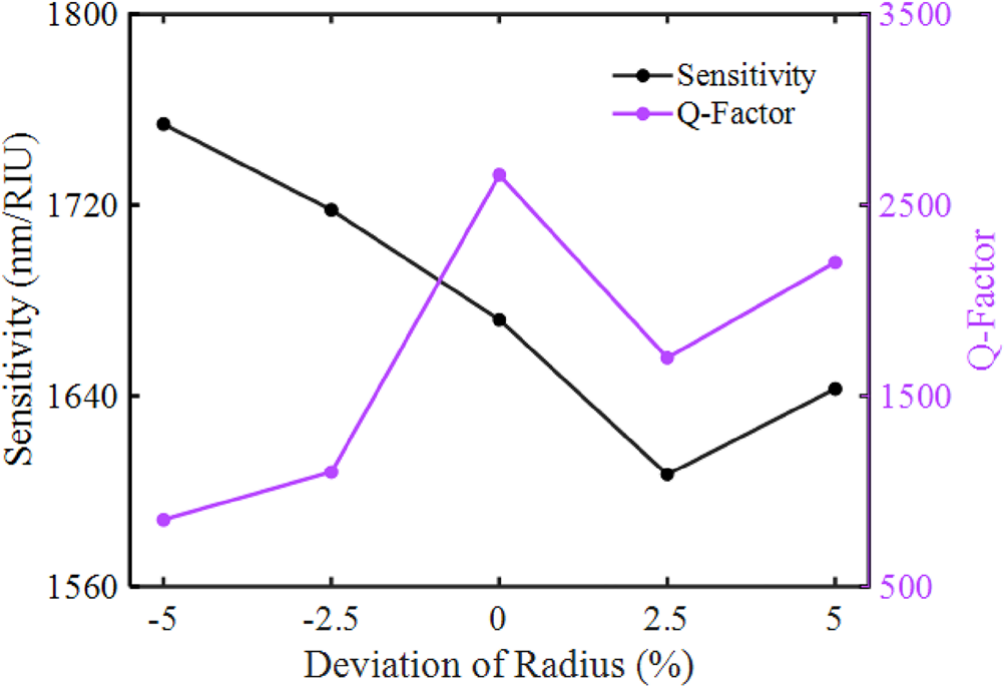
The variations of quality factor and sensitivity when photonic crystal and metal rods experience a radius deviation.

Due to deviations which happen in the actual fabrication processes, we analyze the stability of the sensor by examining the effect of changing the radius of all rods on the sensing performance. As shown in Fig. 14, we assume radius deviations for all rods from − 5 to 5% by a 2.5% step. The influence of the radius variation on the sensitivity and the quality factor of the resonance modes in the normalized transmission spectrum are investigated.

In Table 2, the sensing performance of the proposed hybrid P-PhC RI sensor is compared with other RI sensors recently reported in the literature (such as plasmonic-based sensors and PhC-based sensors). According to this table, the proposed hybrid sensor has higher sensitivity and higher FoM values compared to other RI sensors presented in this table. It is well known that the main challenge of many sensors is that both sensitivity and FoM values are not improved simultaneously. We believe that the proposed hybrid P-PhC RI sensor can open up new windows for designing RI sensors with better sensing properties.

**Table 2:** Comparison of the sensing performance of the proposed hybrid PhC-P RI sensor with other RI sensors recently reported in the literature.

Sensor type	Sensitivity (nm/RIU)	FoM (RIU^−1^)	Year	References
Plasmonic sensor	623	93	2016	^69^
Plasmonic sensor	1125	74	2017	^70^
Plasmonic sensor	1700	~ 61	2018	^71^
Plasmonic sensor	1580	105	2019	^72^
Plasmonic sensor	636	~ 211	2019	^12^
Plasmonic sensor	1200	122	2019	^73^
Plasmonic sensor	~ 1241	~ 44	2020	^74^
Plasmonic sensor	1000	~ 287	2020	^33^
Plasmonic sensor	1320	~ 17	2021	^75^
Plasmonic sensor	1050	~ 108	2021	^76^
PhC sensor	~ 388	~ 1050	2016	^77^
PhC sensor	321	~ 770	2017	^78^
PhC sensor	720	–	2018	^20^
PhC sensor	1150	–	2018	^79^
PhC sensor	739	737	2019	^27^
PhC sensor	544	1234	2019	^80^
PhC sensor	270	–	2020	^81^
PhC sensor	508	–	2020	^82^
PhC sensor	~ 387	~ 1174	2021	^83^
PhC sensor	332	–	2021	^28^
Hybrid P-PhC sensor	1350	488	2021	^53^
Hybrid P-PhC sensor	1250	2083	2022	^5^
Hybrid P-PhC sensor	1672	2388	2022	This work

## Conclusions

In this paper, an optical refractive index (RI) sensor based on a hybrid plasmonic-photonic crystal (P-PhC) design was proposed. In the proposed design, some metallic rods were placed in the coupling regions between waveguides and the cavity in a Si rod-type PhC structure. The analyte area was considered inside the cavity. This structure increased light-analyte interaction by adding metallic rods in the coupling regions. Numerical simulations were performed based on the FDTD method. The proposed hybrid sensor can simultaneously enhance sensitivity and figure of merit (FoM) values. According to the results, a sensitivity of 1672 nm/RIU and a large FoM of 2388 RIU^−1^ were obtained for the hybrid P-PhC mode, which were larger than those of the pure PhC sensor structures without metallic rods, respectively. Therefore, the proposed hybrid P-PhC RI sensor can be a more fascinating candidate for high-sensitivity and high-resolution sensing applications at optic communication wavelengths.

## Data availabiltiy

The datasets generated and analyzed during the current study are available from the corresponding author on reasonable request.

## References

[1] El Shamy RS, Swillam MA, Li X (2022). On-chip complex refractive index detection at multiple wavelengths for selective sensing. Sci. Rep..

[2] White IM, Fan X (2008). On the performance quantification of resonant refractive index sensors. Opt. Express.

[3] Anker, J. N., Hall, W. P., Lyandres, O., Shah, N. C., Zhao, J., Van Duyne, R. P. Biosensing with plasmonic nanosensors. Nanosci. Technol. Collection of Reviews from Nature Journals, 308–319 (2010).

[4] Špačková B, Wrobel P, Bocková M, Homola J (2016). Optical biosensors based on plasmonic nanostructures: A review. Proc. IEEE.

[5] Hajshahvaladi L, Kaatuzian H, Danaie M (2022). A very high-resolution refractive index sensor based on hybrid topology of photonic crystal cavity and plasmonic nested split-ring resonator. Photonics Nanostruct. Fundam. Appl..

[6] Mayer KM, Hafner JH (2011). Localized surface plasmon resonance sensors. Chem. Rev..

[7] Hajshahvaladi L, Kaatuzian H, Danaie M, Karimi Y (2022). Design of a highly sensitive tunable plasmonic refractive index sensor based on a ring-shaped nano-resonator. Opt. Quantum Electron..

[8] Hajshahvaladi L, Kaatuzian H, Danaie M (2021). A high-sensitivity refractive index biosensor based on Si nanorings coupled to plasmonic nanohole arrays for glucose detection in water solution. Opt. Commun..

[9] Wang A, Dan Y (2018). Mid-infrared plasmonic multispectral filters. Sci. Rep..

[10] Hajshahvaladi, L., Kaatuzian, H., Danaie, M., Nourbakhsh, G. Realization of a high-resolution plasmonic refractive index sensor based on double-nanodisk shaped resonators. In *2022 30th International Conference on Electrical Engineering* (ICEE), 926–930 (2022).

[11] Atwater HA (2007). The promise of plasmonics. Sci. Am..

[12] Danaie M, Shahzadi A (2019). Design of a high-resolution metal–insulator–metal plasmonic refractive index sensor based on a ring-shaped si resonator. Plasmonics.

[13] Thadson K, Sasivimolkul S, Suvarnaphaet P, Visitsattapongse S, Pechprasarn S (2022). Measurement precision enhancement of surface plasmon resonance based angular scanning detection using deep learning. Sci. Rep..

[14] Nasirifar R, Danaie M, Dideban A (2022). Highly sensitive surface plasmon resonance sensor using perforated optical fiber for biomedical applications. Optik.

[15] Zhou J, Al Husseini D, Li J, Lin Z, Sukhishvili S, Coté GL (2022). Detection of volatile organic compounds using mid-infrared silicon nitride waveguide sensors. Sci. Rep..

[16] Nickpay M-R, Danaie M, Shahzadi A (2022). Design of a graphene-based multi-band metamaterial perfect absorber in THz frequency region for refractive index sensing. Physica E.

[17] Chou Chau Y-F, Ming TY, Chou Chao C-T, Thotagamuge R, Kooh MRR, Huang HJ (2021). Significantly enhanced coupling effect and gap plasmon resonance in a MIM-cavity based sensing structure. Sci. Rep..

[18] Nasirifar R, Danaie M, Dideban A (2021). Surface plasmon resonance biosensor using inverted graded index optical fiber. Photonics Nanostruct. Fundam. Appl..

[19] Nickpay MR, Danaie M, Shahzadi A (2021). Highly sensitive THz refractive index sensor based on folded split-ring metamaterial graphene resonators. Plasmonics.

[20] Danaie M, Kiani B (2018). Design of a label-free photonic crystal refractive index sensor for biomedical applications. Photonics Nanostruct. Fundam. Appl..

[21] Zaky ZA, Ahmed AM, Shalaby AS, Aly AH (2020). Refractive index gas sensor based on the Tamm state in a one-dimensional photonic crystal: Theoretical optimisation. Sci. Rep..

[22] Liu Y, Salemink H (2014). All-optical on-chip sensor for high refractive index sensing in photonic crystals. EPL (Europhysics Letters).

[23] Fenzl C, Hirsch T, Wolfbeis OS (2014). Photonic crystals for chemical sensing and biosensing. Angew. Chem. Int. Ed..

[24] Nohoji AHA, Danaie M (2022). Highly sensitive refractive index sensor based on photonic crystal ring resonators nested in a Mach–Zehnder interferometer. Opt. Quantum Electron..

[25] Lai W-C, Chakravarty S, Zou Y, Chen RT (2012). Silicon nano-membrane based photonic crystal microcavities for high sensitivity bio-sensing. Opt. Lett..

[26] Li T, Zhu L, Yang X, Lou X, Yu L (2020). A refractive index sensor based on H-shaped photonic crystal fibers coated with Ag-graphene layers. Sensors.

[27] Rahman-Zadeh F, Danaie M, Kaatuzian H (2019). Design of a highly sensitive photonic crystal refractive index sensor incorporating ring-shaped GaAs cavity. Opto-Electron. Rev..

[28] Cheng Q, Wang S, Lv J, Wang J, Liu N (2021). A photonic crystal sensor array side-coupled to a linear waveguide with enhanced bandwidth. Opt. Commun..

[29] Farhadi S, Miri M, Farmani A (2021). Plasmon-induced transparency sensor for detection of minuscule refractive index changes in ultra-low index materials. Sci. Rep..

[30] Kaatuzian, H. & Taheri, A. N. Applications of nano-scale plasmonic structures in design of stub filters—A step towards realization of plasmonic switches. In *Photonic Crystals*, ed: BoD–Books on Demand 93 (2015).

[31] Cetin AE, Topkaya SN (2019). Photonic crystal and plasmonic nanohole based label-free biodetection. Biosens. Bioelectron..

[32] Barnes WL, Dereux A, Ebbesen TW (2003). Surface plasmon subwavelength optics. Nature.

[33] Butt MA, Kazanskiy NL, Khonina SN (2020). Modal characteristics of refractive index engineered hybrid plasmonic waveguide. IEEE Sens. J..

[34] Jiang M, Qi J, Zhang M, Sun Q, Chen J, Chen Z (2017). Ultra-high quality factor metallic micro-cavity based on concentric double metal-insulator-metal rings. Sci. Rep..

[35] Hajshahvaladi L, Kaatuzian H, Danaie M (2019). Design and analysis of a plasmonic demultiplexer based on band-stop filters using double-nanodisk-shaped resonators. Opt. Quantum Electron..

[36] Rashed A, Gudulluoglu B, Yun H, Habib M, Boyaci I, Hong S (2018). Highly-sensitive refractive index sensing by near-infrared metatronic nanocircuits. Sci. Rep..

[37] Abasahl B, Santschi C, Raziman T, Martin OJ (2021). Fabrication of plasmonic structures with well-controlled nanometric features: A comparison between lift-off and ion beam etching. Nanotechnology.

[38] Hajshahvaladi L, Kaatuzian H, Danaie M (2017). Design and simulation of infrared a photonic crystal band pass filters for fiber optics communication. Iran. Conf. Electr. Eng..

[39] Yasumoto K (2018). Electromagnetic Theory and Applications for Photonic Crystals.

[40] Hajshahvaladi L, Kaatuzian H, Danaie M (2017). Analysis and design of semiconductor photonic crystal double bandpass filter for CWDM systems. Int. J. Opt. Appl..

[41] Petrova I, Konopsky V, Nabiev I, Sukhanova A (2019). Label-free flow multiplex biosensing via photonic crystal surface mode detection. Sci. Rep..

[42] Sakoda K (2004). Optical Properties of Photonic Crystals.

[43] Liu Z, Sun F, Wang C, Tian H (2019). Side-coupled nanoscale photonic crystal structure with high-Q and high-stability for simultaneous refractive index and temperature sensing. J. Mod. Opt..

[44] Wang J, Pinkse PW, Segerink LI, Eijkel JC (2021). Bottom-up assembled photonic crystals for structure-enabled label-free sensing. ACS Nano.

[45] Hajshahvaladi, L., Kaatuzian, H., Danaie, M., & Nohiji, A. A. The effect of metal rods in a hybrid plasmonic-photonic crystal cavity design. In *2022 30th International Conference on Electrical Engineering* (ICEE), 936–940 (2022).

[46] Chiang, C.-K., Chung, Y.-C., Cheng, P.-J., Wu, C.-W., Chang, S.-W., & Lin, T.-R. High Q/Vm hybrid photonic-plasmonic crystal nanowire cavity at telecommunication wavelengths. In *Physics and Simulation of Optoelectronic Devices* XXIII 281–286 (2015).

[47] Yu X, Shi L, Han D, Zi J, Braun PV (2010). High quality factor metallodielectric hybrid plasmonic–photonic crystals. Adv. Func. Mater..

[48] Shaban M, Ahmed AM, Abdel-Rahman E, Hamdy H (2017). Tunability and sensing properties of plasmonic/1D photonic crystal. Sci. Rep..

[49] Paternò GM, Moscardi L, Donini S, Ariodanti D, Kriegel I, Zani M (2019). Hybrid one-dimensional plasmonic–photonic crystals for optical detection of bacterial contaminants. J. Phys. Chem. Lett..

[50] Xu Y, Bai P, Zhou X, Akimov Y, Png CE, Ang LK (2019). Optical refractive index sensors with plasmonic and photonic structures: Promising and inconvenient truth. Adv. Opt. Mater..

[51] Barth M, Schietinger S, Fischer S, Becker J, Nusse N, Aichele T (2010). Nanoassembled plasmonic-photonic hybrid cavity for tailored light-matter coupling. Nano Lett..

[52] De Angelis F, Patrini M, Das G, Maksymov I, Galli M, Businaro L (2008). A hybrid plasmonic—Photonic nanodevice for label-free detection of a few molecules. Nano Lett..

[53] Hajshahvaladi L, Kaatuzian H, Danaie M (2021). Design of a hybrid photonic-plasmonic crystal refractive index sensor for highly sensitive and high-resolution sensing applications. Phys. Lett. A.

[54] Lin S, Crozier KB (2013). Trapping-assisted sensing of particles and proteins using on-chip optical microcavities. ACS Nano.

[55] Khani S, Hayati M (2022). Optical biosensors using plasmonic and photonic crystal band-gap structures for the detection of basal cell cancer. Sci. Rep..

[56] Zhang T, Callard S, Jamois C, Chevalier C, Feng D, Belarouci A (2014). Plasmonic-photonic crystal coupled nanolaser. Nanotechnology.

[57] Danaie M, Geravand A, Mohammadi S (2018). Photonic crystal double-coupled cavity waveguides and their application in design of slow-light delay lines. Photonics Nanostruct. Fundam. Appl..

[58] Liu Z, Yu M, Huang S, Liu X, Wang Y, Liu M (2015). Enhancing refractive index sensing capability with hybrid plasmonic–photonic absorbers. J. Mater. Chem. C.

[59] Geravand A, Danaie M, Danaee E (2020). Low cross-talk waveguide intersections for TE polarization using photonic crystals. Opt. Commun..

[60] Shafagh SG, Kaatuzian H, Danaie M (2021). Ahighly sensitive tunable filter using hybrid 1-D photonic crystal and plasmonic MIM waveguide. Optik.

[61] Kyoung J, Kang HE, Hwang SW (2017). Surface plasmonometry: high-resolution and model-free plasmonic measurements of the refractive index and its biosensing application. ACS Photonics.

[62] Pozar DM (2009). Microwave Engineering USA.

[63] Oka H, Ohdaira Y (2018). Simple model of saturable localised surface plasmon. Sci. Rep..

[64] Du L, Zhang X, Mei T, Yuan X (2010). Localized surface plasmons, surface plasmon polaritons, and their coupling in 2D metallic array for SERS. Opt. Express.

[65] Maier SA (2007). Plasmonics: Fundamentals and Applications.

[66] Waks E, Vuckovic J (2005). Coupled mode theory for photonic crystal cavity-waveguide interaction. Opt. Express.

[67] Johnson PB, Christy R-W (1972). Optical constants of the noble metals. Phys. Rev. B.

[68] Joannopoulos JD, Johnson SG, Winn JN, Meade RD (2011). Photonic Crystals: Molding the Flow of Light.

[69] Liang Y, Lu M, Chu S, Li L, Peng W (2016). Tunable plasmonic resonances in the hexagonal nanoarrays of annular aperture for biosensing. Plasmonics.

[70] Tang Y, Zhang Z, Wang R, Hai Z, Xue C, Zhang W (2017). Refractive index sensor based on fano resonances in metal-insulator-metal waveguides coupled with resonators. Sensors.

[71] Yi X, Tian J, Yang R (2018). Tunable fano resonance in plasmonic MDM waveguide with a square type split-ring resonator. Optik.

[72] Chen Z, Li P, Zhang S, Chen Y, Liu P, Duan H (2019). Enhanced extraordinary optical transmission and refractive-index sensing sensitivity in tapered plasmonic nanohole arrays. Nanotechnology.

[73] Zhang Y, Kuang Y, Zhang Z, Tang Y, Han J, Wang R (2019). High-sensitivity refractive index sensors based on Fano resonance in the plasmonic system of splitting ring cavity-coupled MIM waveguide with tooth cavity. Appl. Phys. A.

[74] Alipour A, Mir A, Farmani A (2020). Ultra high-sensitivity and tunable dual-band perfect absorber as a plasmonic sensor. Opt. Laser Technol..

[75] Butt M, Khonina S, Kazanskiy N (2021). Metal-insulator-metal nano square ring resonator for gas sensing applications. Waves Random Complex Media.

[76] Khani S, Hayati M (2021). Optical sensing in single-mode filters base on surface plasmon H-shaped cavities. Opt. Commun..

[77] Jindal S, Sobti S, Kumar M, Sharma S, Pal MK (2016). Nanocavity-Coupled Photonic Crystal Waveguide as Highly Sensitive Platform for Cancer Detection. IEEE Sens. J..

[78] Liu W, Yan J, Shi Y (2017). High sensitivity visible light refractive index sensor based on high order mode Si 3 N 4 photonic crystal nanobeam cavity. Opt. Express.

[79] Kassa-Baghdouche L, Cassan E (2018). Mid-infrared refractive index sensing using optimized slotted photonic crystal waveguides. Photonics Nanostruct. Fundam. Appl..

[80] Olyaee S, Seifouri M, Karami R, Mohebzadeh-Bahabady A (2019). Designing a high sensitivity hexagonal nano-cavity photonic crystal resonator for the purpose of seawater salinity sensing. Opt. Quantum Electron..

[81] Kassa-Baghdouche L, Cassan E (2020). Mid-infrared gas sensor based on high-Q/V point-defect photonic crystal nanocavities. Opt. Quantum Electron..

[82] Fu Y-L, Deng C-S, Ma S-S (2020). Design and analysis of refractive index sensors based on slotted photonic crystal nanobeam cavities with sidewall gratings. Appl. Opt..

[83] Panda A, Pukhrambam PD (2021). Investigation of defect based 1D photonic crystal structure for real-time detection of waterborne bacteria. Physica B: Condens. Matter..

